# Organic Selenium Increased Gilts Antioxidant Capacity, Immune Function, and Changed Intestinal Microbiota

**DOI:** 10.3389/fmicb.2021.723190

**Published:** 2021-08-16

**Authors:** Zimei Li, Yanpeng Dong, Sirun Chen, Xinlin Jia, Xuemei Jiang, Lianqiang Che, Yan Lin, Jian Li, Bin Feng, Zhengfeng Fang, Yong Zhuo, Jianping Wang, Haitao Xu, De Wu, Shengyu Xu

**Affiliations:** ^1^Animal Nutrition Institute, Key Laboratory of Animal Disease-resistant Nutrition, Ministry of Education, Ministry of Agriculture and Rural Affairs, Sichuan Agricultural University, Chengdu, China; ^2^Animal Husbandry Development Center of Changyi City, Shandong, China

**Keywords:** 2-hydroxy-4-methylselenobutanoic acid, intestinal microbiota, gilts, antioxidant capacity, immune function

## Abstract

Selenium is an indispensable essential micronutrient for humans and animals, and it can affect biological functions by combining into selenoproteins. The purpose of this study was to investigate the effects of 2-hydroxy-4-methylselenobutanoic acid (HMSeBA) on the antioxidant performance, immune function, and intestinal microbiota composition of gilts. From weaning to the 19th day after the second estrus, 36 gilts (Duroc × Landrace × Yorkshire) were assigned to three treatments: control group, sodium selenite group (0.3 mg Se/kg Na_2_SeO_3_), and HMSeBA group (0.3 mg Se/kg HMSeBA). Dietary supplementation with HMSeBA improved the gilts tissue selenium content (except in the thymus) and selenoprotein P (SelP1) concentration when compared to the Na_2_SeO_3_ or control group. Compared with the control group, the antioxidant enzyme activity in the tissues from gilts in the HMSeBA group was increased, and the concentration of malondialdehyde in the colon had a decreasing trend (*p* = 0.07). Gilts in the HMSeBA supplemented group had upregulated gene expression of *GPX2*, *GPX4*, and *SelX* in spleen tissue, *TrxR1* in thymus; *GPX1* and *SelX* in duodenum, *GPX3* and *SEPHS2* in jejunum, and *GPX1* in the ileum tissues (*p* < 0.05). In addition, compared with the control group, the expression of *interleukin-1β* (*IL-1β*), *interleukin-6* (*IL-6*), *interleukin-8* (*IL-8*), and *monocyte chemotactic protein-1* (*MCP-1*) in the liver, spleen, thymus, duodenum, ileum, and jejunum of gilts in the HMSeBA group were downregulated (*p* < 0.05), while the expression of *interleukin-10* (*IL-10*) and *transforming growth factor-β* (TGF*-β*) in the liver, thymus, jejunum, and ileum were upregulated (*p* < 0.05). Compared with the control group and the Na_2_SeO_3_ group, HMSeBA had increased concentration of serum cytokines interleukin-2 (IL-2) and immunoglobulin G (IgG; *p* < 0.05), increased concentration of intestinal immunoglobulin A (sIgA; *p* < 0.05), and decreased concentration of serum IL-6 (*p* < 0.05). Dietary supplementation with HMSeBA also increased the abundance of intestinal bacteria (*Ruminococcaceae* and *Phascolarctobacterium*; *p* < 0.05) and selectively inhibited the abundance of some bacteria (*Parabacteroides* and *Prevotellaceae*; *p* < 0.05). In short, HMSeBA improves the antioxidant performance and immune function of gilts, and changed the structure of the intestinal microflora. And this study provided data support for the application of HMSeBA in gilt and even pig production.

## Introduction

As an essential micronutrient, selenium plays an important biological role in animals and human body by participating in the composition of selenocysteine and selenoprotein (Schwarz and Fredga, 1969). Selenium is known to be the main component of 25 selenoproteins, most of which have antioxidant and immune functions (Schrauzer and Gerhard, 2000). Due to many selenoproteins having antioxidant activity, selenium has long been considered to protect the body and intestine from inflammation by reducing oxidative damage. Selenium is also an important component of glutathione peroxidase (GPH-Px) and thioredoxin reductase (TrxR) in animals, and plays an important role in antioxidation and immunity (Stadtman, 1996; Costello, 2001; Hawkes and Alkan, 2010). Studies have found that selenium deficiency has adverse effects on the growth, reproduction, and immune function of animals (Żarczyńska et al., 2013). Adding selenium and selenium products to the basic diet of animals can promote growth, improve immune function, and reduce oxidative stress (Cao et al., 2015). Margarida et al. (2020) added sodium selenite or selenium-enriched yeast to the diet of puppies and found that organic selenium reduced the DNA concentration of *Escherichia coli*, increased the DNA concentration of lactic acid bacteria, and increased the concentrations of volatile fatty acids, butyric acid, and propionic acid in puppies, which was conducive to the intestinal immunity of puppies (Margarida et al., 2020). Therefore, adding selenium to the basic diet of animals can improve immune function and change the intestinal microflora.

In animal diets, selenium mainly exists either as inorganic or organic forms. Many studies have shown that organic selenium (methionine selenium, selenium yeast, selenium enriched probiotics, etc.) has less toxicity and higher biological potency than inorganic selenium (sodium selenite and sodium selenate, etc.; Vendeland et al., 1994; Alimohamady et al., 2013; Rita and Nancy, 2015). A new organic selenium source, 2-hydroxy-4-methylselenobutanoic acid (HMSeBA) with a selenium content of 2%, is much higher than other organic selenium sources. The effectiveness of this new organic selenium source in poultry, growing pigs, and sows has been reported (Jlali et al., 2013; Chao et al., 2019). Adding HMSEBA in the diet of sows can improve the antioxidant capacity of sows and their offspring (Mou et al., 2020a). However, the effect of HMSeBA on gilts has not been extensively studied. Therefore, the purpose of this study was to investigate the effects of HMSeBA as a feed additive on the antioxidant capacity, immune function, and intestinal microbiota of gilts.

## Materials and Methods

All procedures involving animals in this study were approved by the Animal Care and Use Committee of Sichuan Agricultural University (Approval number: 20200722).

### Animal and Experimental Designs

A total of 36 gilts (Duroc × Landrace × Yorkshire) with similar body weight (BW; initial body weight 5.50 ± 0.09 kg) were assigned to three treatment groups: (1) control diet (gilts were fed a basic diet from weaning to the 19th day after the second estrus, *n* = 12), (2) sodium selenite (Na_2_SeO_3_) supplemented diet (Na_2_SeO_3_, basal diet + Na_2_SeO_3_ at 0.3 mg Se/kg, *n* = 12), and (3) HMSeBA supplemented diet (HMSeBA, basal diet + HMSeBA at 0.3 mg Se/kg, *n* = 12). The powdered basal diet is presented in Table 1, which was formulated according to the nutrient requirements recommended by the National Research Council (2012) except for that of selenium. The dietary Se level was formulated with Na_2_SeO_3_ and HMSeBA according to the experimental design shown in Table 2. The selenium additive is added to the gilt diet in the form of a premix. And the 2-hydroxy-4-methylselenobutanoic acid (HMSeBA, Selisseo® 2% Se) was provided by Adisseo France S.A.S, and Na_2_SeO_3_ was provided by Chengdu Shuxing Feed Co. Ltd. (1% Se).

**Table 1:** Composition and nutrient levels of the basal diet (as-fed basis).

	7–25 kg	25–75 kg	75 kg-end
**Ingredient, %**			
De-hulled soybean meal, 46% CP	15.00	-	-
Extruded maize meal, 8.24% CP	12.40	-	-
Expanded soybean, 35.5% CP	10.00	-	-
Whey powder, 2% CP	5.00	-	-
Sucrose	3.90	-	-
Corn, 8.24% CP	45.00	69.48	72.00
Soybean, 44% CP	-	19.00	14.00
Wheat bran, 15% CP	-	5.00	7.57
Fish meal, 62.5% CP	3.00	0.50	1.50
Soybean oil	2.00	2.00	2.00
_L_-Lys HCl, 98%	0.60	0.49	0.28
_DL_-Met, 98.5%	0.22	0.10	0.04
_L_-Thr, 98%	0.19	0.17	0.08
_L_-Trp, 98%	0.05	0.05	0.03
Choline chloride, 50%	0.16	0.16	0.12
Limestone	0.93	0.94	1.00
CaHPO_4_	0.94	1.70	1.00
Sodium chloride	0.40	0.28	0.25
Mineral premix	0.32^1^	0.20^2^	0.20^2^
Vitamin premix	0.05^3^	0.03^4^	0.03^4^
Total	100.00	100.00	100.00
**Nutrient level** ^5^			
Digestible energy, Mcal/kg	3.538	3.449	3.430
Crude protein, %	17.06	14.21	14.52
Ca, %	0.85	0.85	0.79
Total P, %	0.52	0.51	0.52
Available P, %	0.34	0.42	0.32
SID Lys, %	1.36	1.01	0.78
SID Met, %	0.50	0.33	0.27
SID Met+Cys, %	0.68	0.55	0.48
SI Thr, %	0.78	0.61	0.49
SID Trp, %	0.22	0.19	0.15

1 *Per kilogram of diet provided: 125 mg Fe; 14 mg Cu; 30 mg Mn; 110 mg Zn; 0.30 mg I*.

2 *Per kilogram of diet provided: 120 mg Fe; 12 mg Cu; 30 mg Mn; 100 mg Zn; 0.28 mg I*.

3 *Per kilogram of diet provided: 12,000 IU VA; 2,400 IU VD3; 100 IU VE; 4.8 mg VK3; 2 mg VB1; 7.2 mg VB2; 3.6 mg VB6; 0.025 mg VB12; 0.48 mg biotin; 25 mg pantothenic acid; 4 mg folic acid; 40 mg niacin*.

4 *Per kilogram of diet provided: 7,200 IU VA; 1,440 IU VD3; 60 IU VE; 2.88 mg VK3; 1.2 mg VB1; 4.32 mg VB2; 2.16 mg VB6; 0.015 mg VB12; 0.288 mg biotin; 15 mg pantothenic acid; 2.4 mg folic acid; 24 mg niacin*.

5 *Except for the crude protein, total Ca, and P are measured values, the rest are calculated*.

**Table 2:** Selenium sources and levels in the diets.

Treatment	Diet	Supplemented Se (mg/kg)	Total Se content (mg/kg)^a^		
			5–25 kg	25–75 kg	75 kg above
Control	Basal diet	0.00	0.09	0.06	0.04
Na_2_SeO_3_	Basal diet + 0.3 mg Se/kg Na_2_SeO_3_	0.30	0.35	0.33	0.31
HMSeBA	Basal diet + 0.3 mg Se/kg HMSeBA	0.30	0.37	0.41	0.41

*HMSeBA, 2-hydroxy-4-methylselenobutanoic acid; Se Selenium*.

a *Analysed values*.

During the experiment, gilts were fed four times a day from weaning to 90 days of age (08:00; 12:00; 16:00; 20:00), and from 90 days to slaughter, they were fed twice a day (08: 00; 16:00). Up to 176 days of age, the gilts were fed freely; 176 days of age to slaughter, the daily feeding limit was 2.5 kg. After 180 days of age, boars were used to check for estrus twice a day (08:00; 16:00). Observation of the vulva and back pressure reflex was used to determine estrus response. On the 19th day of the third estrus period, five gilts were randomly selected from each treatment group for slaughter.

### Sample Collection

Before slaughter, gilts were fasted 12 h and 5 ml of blood was collected from the anterior vena cava of the gilts. After standing for 20 min, the blood samples were centrifuged at 2,800 r/min at 4°C for 20 min. Following centrifugation serum was collected in a centrifuge tube, and stored at −20°C for further analysis.

After the gilt was slaughtered, the abdominal cavity was opened immediately, the intestines were taken out, and each intestine segment was separated. After the duodenum, jejunum, and ileum were rinsed with normal saline (0.9% NaCl), the intestinal mucosa from a 6 cm middle area was scraped off using a slide and snap-frozen in liquid nitrogen, and a 2 cm middle tissue sample was rinsed and then snap frozen in liquid nitrogen. The colonic chyme was snap-frozen in liquid nitrogen and stored at −80°C for further analysis (Wan et al., 2020).

The liver, spleen, and thymus were excised and weighed. Then the samples from liver, spleen, and thymus were rinsed in ice-cold saline (0.9% NaCl), snap frozen in liquid nitrogen, and stored at −80°C for further analysis (Mou et al., 2018).

### Biochemical Analysis

#### Sample Preparation

The frozen tissue samples were thawed and placed in a sterile test tube containing 2 ml ice-cold PBS. Tissue specimen (g): PBS (ml) = 1:9. The mixture of the weighed tissue sample and ice-cold PBS was homogenized with a tissue homogenizer (bullet mixer). After centrifugation at 6,000 r/min 4°C for 15 min, the supernatant was divided into 200 μl sterile tubes and stored at −80°C for testing (Mou et al., 2018, 2020b).

#### Determination of Selenium Content

For the determination of selenium content in feed, tissue, and serum samples, we referred to the “National Food Safety Standard Determination of Selenium in Food” (GB5009.93-2017). Briefly, weighed about 1 g/1 ml of sample and added acid (nitric acid: perchloric acid = 4:1) for overnight digestion at room temperature, following digestion, the samples were heated to 365°C until the solution became clear and colorless. We then used hydrochloric acid and potassium borohydride to reduce the hexavalent selenium in the sample to hydrogen selenide in a hydrochloric acid medium. Subsequently, the total selenium content in the sample was determined by hydride atomic fluorescence spectrometry (AFS-9230, Beijing Auspicious Day Instrument Co., Ltd., Beijing, China).

#### Antioxidant Determination

The activities of antioxidant GPH-Px (Serial No. A005-1-2), catalase (CAT, Serial No. A064-1-1), total superoxide dismutase (T-SOD, Serial No. A001-1-2), total antioxidant capacity (T-AOC, Serial No. A015-2-1), glutathione reductase (GR, Serial No. A062-1-1), and the concentration of malondialdehyde (MDA, Serial No. A003-1-2) were analyzed using the corresponding commercial assay kit (Nanjing Institute of Jiancheng Biological Engineering, Nanjing, China) according to the manufacturer’s instructions. The antioxidant activity of the above parameters was calculated based on the protein content of the tissue sample, and the assay was performed using the method described by Bradford (1976).

#### Serum, Intestinal Biochemical Indicators

Serum interleukin-2 (IL-2, Serial No. H003) and interleukin-6 (IL-6, Serial No. H007-1-1), immunoglobulin A (IgA, Serial No. H108-1), immunoglobulin G (IgG, Serial No. H106), immunoglobulin M (IgM, Serial No. H109), tumor necrosis factor-α (TNF-α, Serial No. H052-1), and intestinal immunoglobulin A (sIgA, Serial No. H108-2) concentrations were determined using ELISAs and the corresponding commercial assay kits (Nanjing Jiancheng Bioengineering Institute) according to the manufacturer’s instructions.

#### Total RNA Extraction and Real-Time RT-PCR

Trizol reagent was used to extract total RNA from the frozen tissue samples. Agarose gel electrophoresis was used to check RNA integrity. RNA purity was checked using a nucleic acid/protein analyzer. A PrimeScript RT kit with gDNA eraser was used to perform genomic DNA removal and reverse transcription (RT) according to the manufacturer’s instructions. A SYBR Premix Ex TaqTM kit was used for real-time PCR analysis of mRNA transcript expression. The PCR protocol was 1 cycle of 95°C for 30 s and 40 cycles of 95°C for 15 s followed by 60°C for 1 min. At the end of amplification, melting curve analysis was performed to verify specific amplifications using an ABI-7900HT Fast Real-Time PCR System (Applied Biosystems, CA, United States). Real-time PCR data were analyzed by 2-delta CT method with β-actin as the reference gene (Livak and Schmittgen, 2001) Table 3 shows the primer sequences of individual genes (Fossum et al., 2014; Mou et al., 2020b).

**Table 3:** Primer sequences of the target and reference genes.

Genes	Primer	Sequence (5'→3')	Accession no.
*SelP 1*	Forward	AACCAGAAGCGCCAGACACT	EF113596
	Reverse	TGCTGGCATATCTCAGTTCTCAGA	
*GPX1*	Forward	GATGCCACTGCCCTCATGA	AF532927
	Reverse	TCGAAGTTCCATGCGATGTC	
*GPX2*	Forward	AGAATGTGGCCTCGCTCTGA	DQ898282
	Reverse	GGCATTGCAGCTCGTTGAG	
*GPX3*	Forward	TGCACTGCAGGAAGAGTTTGAA	AY368622
	Reverse	CCGGTTCCTGTTTTCCAAATT	
*GPX4*	Forward	TGAGGCAAGACGGAGGTAAACT	NM_214407
	Reverse	TCCGTAAACCACACTCAGCATATC	
*TrxR1*	Forward	GATTTAACAAGCGGGTCATGGT	AF537300
	Reverse	CAACCTACATTCACACACGTTCCT	
*TrxR2*	Forward	TCTTGAAAGGCGGAAAAGAGAT	GU181287
	Reverse	TCGGTCGCCCTCCAGTAG	
*SelK*	Forward	CAGGAAACCCCCCTAGAAGAA	NM_001044553.1
	Reverse	CTCATCCACCGGCCATTG	
*SelS*	Forward	GAGGCAGAGGCACCTGGAT	NM_001164113.1
	Reverse	CTGCTAAAGCCTCCTGTCGTTT	
*SelX*	Forward	ATCCCTAAAGGCCAAGAATCATC	EF113597
	Reverse	GGCCACCAAGCAGTGTTCA	
*SEPHS2*	Forward	TGGCTTGATGCACACGTTTAA	NM_001093735
	Reverse	TGCGAGTGTCCCAGAATGC	
*IL-1β*	Forward	TCTGCCCTGTACCCCAACTG	NM_214055.1
	Reverse	CCAGGAAGACGGGCTTTTG	
*IL-6*	Forward	ATGCTTCCAATCTGGGTTCAA	NM_001252429.1
	Reverse	CACAAGACCGGTGGTGATTCT	
*IL-8*	Forward	GCAAGAGTAAGTGCAGAACTTCGA	NM_213867.1
	Reverse	GGGTGGAAAGGTGTGGAATG	
*IL-10*	Forward	CAGATGGGCGACTTGTTGCT	NM_214041.1
	Reverse	GGCAACCCAGGTAACCCTTAA	
*TNF-α*	Forward	CGACTCAGTGCCGAGATCAA	NM_214022.1
	Reverse	CCTGCCCAGATTCAGCAAAG	
*TGF-β*	Forward	AGGACCTGGGCTGGAAGTG	NM_214015.1
	Reverse	GGGCCCCAGGCAGAAAT	
*IFN-β*	Forward	TCTCTAGCACTGGCTGGAATGA	JN391525.1
	Reverse	CTGCCCATCAAGTTCCACAA	
*ICAM-1*	Forward	GGAGGTGCTGAAATCTCAATGTG	NM_213816.1
	Reverse	ACCTTCATGGAGCCTCCTTTG	
*MCP-1*	Forward	GCAAGTGTCCTAAAGAAGCAGTGA	NM_214214.1
	Reverse	GCTTGGGTTCTGCACAGATCT	
*INOS-2*	Forward	AGAGCCAGAAGCGCTATCATG	NM_001143690.1
	Reverse	CCCACTGCCCCCTCCTT	
*β-actin*	Forward	TCTGGCACCACACCTTCT	DQ178122
	Reverse	TGATCTGGGTCATCTTCTCAC	

*SelP1, selenoprotein P; GPX, glutathione peroxidase; TrxR, thioredoxin reductase; SelK, selenoprotein K; SelS, selenoprotein S; SelX, selenoprotein X; SEPHS2, selenophosphate synthetase 2; IL-1β, interleukin-1β; IL-6, interleukin-6; IL-8, interleukin-8; IL-10, interleukin-10; TNF-α, tumor necrosis factor-α; TGF-β, transforming growth factor-β; IFN-β, interferon-β; ICAM-1, intercellular cell adhesion molecule-1; MCP-1, monocyte chemotactic protein-1; INOS-2, inducible nitric oxide synthase-2*.

#### Bacterial Community Analysis

The microbial DNA of colon chyme samples was extracted using the Mo Bio Power-Fecal™ DNA Isolation Kit (MO BIO Laboratories, Carlsbad, CA, United States). A nucleic ac-id/protein analyzer (Beckman DU-800, Beckman Coulter, Inc., CA, United States) was used to determine the concentration and purity of the DNA. The DNA samples were sent to a commercial service provider (Novogene Bioinformatics Technology, Beijing, China) for pairing sequencing on the Illumina HiSeq PE250 platform and bioinformatics analyses. According to the selection of sequencing region, sample was used with forward primer 515F (5'-GTGCCAGCMGCCGCGGTAA-3') and a reverse primer 806R (5'-GGACTACHVGGGTWTCTAAT-3') to perform PCR to amplify the V4 hypervariable region of the 16S rRNA gene was used as described previously (Xu et al., 2020).

Use FLASH (V1.2.7, http://ccb.jhu.edu/software/FLASH/) to splice the reads of each sample, the resulting stitched sequence is raw tags data. Reference Qiime (V1.7.0, http://qiime.org/scripts/split_libraries_fastq.html) tags quality control process, get high quality tags data, and then remove the chimera, get the final effective data (Xu et al., 2020). Clustered into OTUs utilizing Uparse v7.0.1001^1^ at 97% sequence similarity. The Ribosomal Database Project (RDP) classifier Version 2.2^2^ was applied to assign taxonomy for 16S rRNA gene sequences. Species annotation was carried out on the OTUs representative sequences, and species annotation analysis was carried out using Mothur method and the SSUrNA database of SILVA^3^ (set a threshold of 0.8–1) to obtain taxonomic information. A Venn diagram was generated for comparison among the OTUs of the treatments. For intestinal microbiota alpha diversity values for each sample were assessed by Qiime 1.7.0. Spearman correlation analysis was used to analyze the mutual change relationship between serum cytokines and microorganisms, and the correlation and significance between them were obtained.

### Statistical Analysis

The data were analyzed using a one-way ANOVA procedure of Statistical Product and Service Solutions (SAS) statistical software (V9.4, SAS Institute Inc., Cary, NC, United States) followed by Tukey’s multiple range test. The results were presented as the mean values with pooled SEM. Differences at *p* < 0.05 were considered to be statistically significant, whereas a tendency was considered when 0.05 ≤ *p* < 0.10.

## Results

### Organic Selenium Increased Selenium Content and Selenoprotein Gene Expression

The results showed that adding HMSeBA to the basal diet of gilts significantly increased the total selenium content in the serum, liver, spleen, duodenum, jejunum, ileum, and colon compared with the control group (*p* < 0.05; Table 4). Na_2_SeO_3_ group increased the total selenium content in liver and colon compared with the control group (*p* < 0.05). In addition, there was no difference in total selenium content in thymus between the Na_2_SeO_3_ group and HMSeBA group compared with the control group (Table 4).

**Table 4:** HMSeBA effect on serum and tissue total selenium content of gilts.

Item	Treatment			Values of *p*
	Control	Na_2_SeO_3_	HMSeBA	
Serum (mg/L)	0.18 ± 0.00^b^	0.21 ± 0.00^a^	0.22 ± 0.01^a^	0.02
Liver (mg/kg)	0.46 ± 0.03^c^	0.67 ± 0.04^b^	0.91 ± 0.02^a^	<0. 01
Spleen (mg/kg)	0.30 ± 0.02^b^	0.29 ± 0.01^b^	0.48 ± 0.04^a^	<0.01
Thymus (mg/kg)	0.21 ± 0.00	0.35 ± 0.07	0.29 ± 0.01	0.24
Duodenum (mg/kg)	0.21 ± 0.02^b^	0.23 ± 0.01^b^	0.30 ± 0.01^a^	<0.01
Jejunum (mg/kg)	0.23 ± 0.00^b^	0.24 ± 0.02^b^	0.32 ± 0.01^a^	<0.01
Ileum (mg/kg)	0.20 ± 0.02^b^	0.24 ± 0.01^b^	0.39 ± 0.05^a^	<0.01
Colon (mg/kg)	0.20 ± 0.01^b^	0.32 ± 0.02^a^	0.31 ± 0.02^a^	<0.01

*Data were shown as means ± SE. n = 5 in each group. Control, basal diet; Na_2_SeO_3_, 0.3 mg/kg Na_2_SeO_3_; HMSeBA, 0.3 mg/kg HMSeBA. ^a,b,c^Mean values within a row with different superscript letters were significantly different (p < 0.05)*.

**Figure 1:**
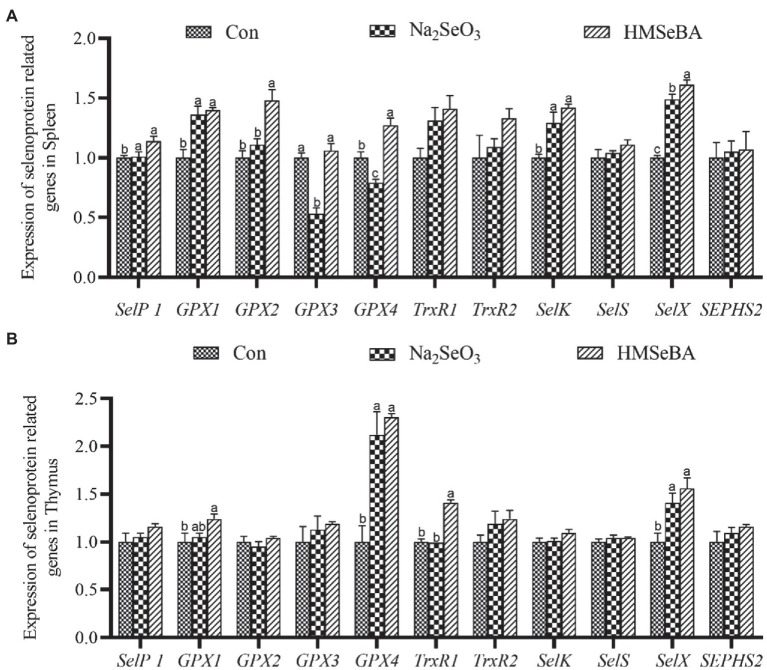
The effect of HMSeBA on the expression of selenoprotein related genes in spleen **(A)** and thymus **(B)** of gilts. *n* = 5 in each group. Data were shown as means ± SE. *n* = 5 in each group. Control, basal diet; Na_2_SeO_3_, 0.3 mg Se/kg Na_2_SeO_3_; HMSeBA, 0.3 mg Se/kg HMSeBA. ^a,b,c^Mean values within a row with different superscript letters were significantly different (*p* < 0.05). SelP1, selenoprotein P; GPX, glutathione peroxidase; TrxR, thioredoxin reductase; SelK, selenoprotein K; SelS, selenoprotein S; SelX, selenoprotein X; SEPHS2, selenophosphate synthetase 2.

The gene expressions of *GPX2*, *GPX4*, and *SelX* in the spleen of gilts added with HMSeBA were increased (*p* < 0.05; Figure 1A) compared with the control group and Na_2_SeO_3_ group. The expressions of *SelP1*, *GPX1*, and *SelK* were higher in HMSeBA and Na_2_SeO_3_ groups than in the control group (*p* < 0.05). In the thymus, the expression of *TrxR1* in the HMSeBA addition group was increased compared with the control and Na_2_SeO_3_ groups (*p* < 0.05), the expression of *GPX1*, *GPX4*, and *SelX* were higher than the control group (*p* < 0.05), but with no difference from the Na_2_SeO_3_ group (Figure 1B).

**Figure 2:**
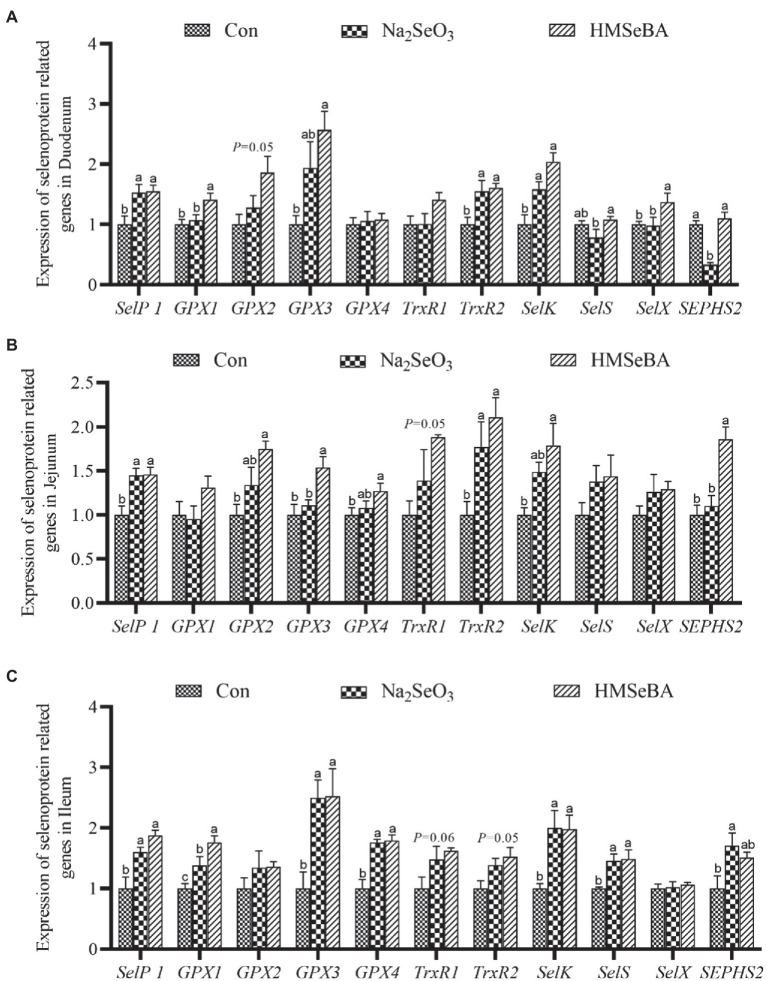
The effect of HMSeBA on the expression of selenoprotein related genes in duodenum **(A)**, jejunum **(B)**, and ileum **(C)** of gilts. *n* = 5 in each group. Data were shown as means ± SE. *n* = 5 in each group. Control, basal diet; Na_2_SeO_3_, 0.3 mg Se/kg Na_2_SeO_3_; HMSeBA, 0.3 mg Se/kg HMSeBA. ^a,b,c^Mean values within a row with different superscript letters were significantly different (*p* < 0.05). SelP1, selenoprotein P; GPX, glutathione peroxidase; TrxR, thioredoxin reductase; SelK, selenoprotein K; SelS, selenoprotein S; SelX, selenoprotein X; SEPHS2, selenophosphate synthetase 2.

The results showed that adding HMSeBA to the basal diet of gilts increased the expression of *GPX1* and *SelX* in the duodenum compared with control and Na_2_SeO_3_ groups (*p* < 0.05), the expressions of *SelP1*, *GPX3*, *TrxR2*, and *SelK* were significantly higher than those in the control group (*p* < 0.05), but no differece with Na_2_SeO_3_ group (Figure 2A). In the jejunum, the expressions of *GPX3* and *SEPHS2* in the HMSeBA addition group were higher than those in the control group and the Na_2_SeO_3_ group (*p* < 0.05), compared with the control group, the expression of *SelP1*, *GPX2*, *GPX4*, *TrxR2*, and *SelK* in the HMSeBA addition group significantly increased (*p* < 0.05). In addition, the expression of *TrxR1* has an increasing trend in HMSeBA group (*p* = 0.05; Figure 2B). The expression of *GPX1* in the ileum of gilts with the HMSeBA containing diet was higher than that of the control group and Na_2_SeO_3_ groups (*p* < 0.05). The expression of *SelP1*, *GPX3*, *GPX4*, *SelK*, and *SelS* in the HMSeBA and Na_2_SeO_3_ groups was higher than those in the control group (*p* < 0.05). The expression of *TrxR1* and *TrxR2* showed an increasing trend (*p* = 0.06, *p* = 0.05) in the HMSeBA group compared with the control group (Figure 2C).

### Organic Selenium Improved the Antioxidant Capacity of Gilts

The results showed that adding HMSeBA to the basal diet of gilts increased the spleen CAT, T-SOD, and GR activity compared with the control group (*p* < 0.05), and there was no significant difference from the Na_2_SeO_3_ group. The activity of GSH-PX in the spleen in the HMSeBA group had an increasing trend (*p* = 0.08) compared with the control group (Table 5). The GR activity of the thymus in the HMSeBA group was increased (*p* < 0.05) compared with the other two groups, the activities of T-AOC, CAT, and GSH-PX were higher than those in the control group (*p* < 0.05), and there was no significant difference from the Na_2_SeO_3_ group. Thymus T-SOD activity in the Na_2_SeO_3_ group was higher than that in the control and HMSeBA groups (*p* < 0.05; Table 5).

**Table 5:** Effect of HMSeBA on antioxidant capacity in gilt tissue.

Item	Treatment			Values of *p*
	Control	Na_2_SeO_3_	HMSeBA	
**Spleen**				
T-AOC (U/mg prot)	0.30 ± 0.02	0.37 ± 0.03	0.32 ± 0.04	0.31
MDA (nmol/mg prot)	2.88 ± 0.30	2.87 ± 0.18	2.63 ± 0.13	0.72
CAT (U/mg prot)	77.17 ± 4.26^b^	87.54 ± 7.75^ab^	102.97 ± 3.75^a^	0.02
T-SOD (U/mg prot)	54.34 ± 11.4^b^	69.33 ± 2.76^a^	69.77 ± 3.02^a^	0.01
GSH-PX (U/mg prot)	30.42 ± 1.35	36.10 ± 1.48	36.49 ± 2.64	0.08
GR (U/g prot)	0.015 ± 0.001^b^	0.019 ± 0.002^ab^	0.026 ± 0.003^a^	0.02
**Thymus**				
T-AOC (U/mg prot)	0.28 ± 0.03^b^	0.45 ± 0.04^a^	0.48 ± 0.04^a^	0.01
MDA (nmol/mg prot)	3.35 ± 0.25	2.98 ± 0.40	3.27 ± 0.26	0.56
CAT (U/mg prot)	34.37 ± 3.76^b^	63.87 ± 4.01^a^	57.34 ± 2.90^a^	<0.01
T-SOD (U/mg prot)	567.83 ± 29.45^c^	797.64 ± 0.77^a^	694.89 ± 20.17^b^	<0.01
GSH-PX (U/mg prot)	20.93 ± 0.23^b^	20.44 ± 0.69^b^	39.39 ± 2.48^a^	<0.01
GR (U/g prot)	0.007 ± 0.002^b^	0.012 ± 0.001^b^	0.017 ± 0.001^a^	<0.01

*Data were shown as means ± SE. n = 5 in each group. Control, basal diet; Na_2_SeO_3_, 0.3 mg Se/kg Na_2_SeO_3_; HMSeBA, 0.3 mg Se/kg g HMSeBA. ^a,b,c^ Mean values within a row with different superscript letters were significantly different (p < 0.05). T-AOC, total antioxidant capacity; MDA, malondialdehyde; CAT, catalase; T-SOD, total superoxide dismutase; GSH-Px, glutathione peroxidase; GR, glutathione reductase*.

Compared with the control group, gilts supplemented with HMSeBA and Na_2_SeO_3_ had increased GR activity in the duodenal (*p* < 0.05; Table 6). The T-SOD activity is decreased in Na_2_SeO_3_ group compared to control group and HMSeBA group. The HMSeBA group had a tendency of increased GSH-PX activity compared with the control group (*p* = 0.06). Compared with the control group and the Na_2_SeO_3_ group, the addition of HMSeBA resulted in increased activities of T-SOD, GSH-PX, and GR in the jejunum (*p* < 0.05). The addition of HMSeBA to the basal diet of gilts increased the activity of GSH-PX and GR in the ileum compared with the control and Na_2_SeO_3_ groups (*p* < 0.05), and had a tendency to increase the activity of T-SOD (*p* = 0.07). The GSH-PX activity in the colon of gilts fed diets with HMSeBA was higher than that in the control and Na_2_SeO_3_ groups (*p* < 0.05). Compared with the control group, the colonic T-AOC, CAT, and GR activities were significantly increased (*p* < 0.05) in the HMSeBA and Na_2_SeO_3_ fed groups. Dietary supplementation with HMSeBA had a tendency to decrease the MDA content (*p* = 0.07; Table 6).

**Table 6:** Effect of HMSeBA on antioxidant capacity of gilt intestinal.

Item	Treatment			Values of *p*
	Control	Na_2_SeO_3_	HMSeBA	
**Duodenum**				
T-AOC (U/mg prot)	0.16 ± 0.01	0.17 ± 0.02	0.17 ± 0.02	0.95
MDA (nmol/mg prot)	2.29 ± 0.18	2.29 ± 0.09	2.03 ± 0.04	0.16
CAT (U/mg prot)	30.43 ± 7.69	48.80 ± 6.09	46.07 ± 5.27	0.15
T-SOD (U/mg prot)	901.42 ± 42.63^a^	738.21 ± 37.55^b^	1020.98 ± 48.62^a^	<0.01
GSH-PX (U/mg prot)	17.42 ± 0.54	20.16 ± 0.82	22.03 ± 1.62	0.06
GR (U/g prot)	0.004 ± 0.001^b^	0.015 ± 0.002^a^	0.018 ± 0.001^a^	<0.01
**Jejunum**				
T-AOC (U/mg prot)	0.17 ± 0.00^a^	0.12 ± 0.01^b^	0.17 ± 0.01^a^	<0.01
MDA (nmol/mg prot)	2.37 ± 0.24	2.15 ± 0.13	2.28 ± 0.04	0.57
CAT (U/mg prot)	16.86 ± 3.22	20.61 ± 2.99	28.83 ± 3.82	0.08
T-SOD (U/mg prot)	564.81 ± 4.31^b^	584.14 ± 4.06^b^	668.43 ± 10.55^a^	0.03
GSH-PX (U/mg prot)	28.23 ± 1.53^b^	11.02 ± 0.92^c^	44.79 ± 2.58^a^	<0.01
GR (U/g prot)	0.012 ± 0.001^b^	0.013 ± 0.001^b^	0.019 ± 0.001^a^	0.02
**Ileum**				
T-AOC (U/mg prot)	0.21 ± 0.01	0.25 ± 0.03	0.23 ± 0.01	0.51
MDA (nmol/mg prot)	2.08 ± 0.10	2.23 ± 0.19	1.84 ± 0.16	0.26
CAT (U/mg prot)	30.26 ± 5.98	37.56 ± 3.31	43.01 ± 8.20	0.43
T-SOD (U/mg prot)	801.08 ± 55.43	777.95 ± 32.28	928.37 ± 46.71	0.07
GSH-PX (U/mg prot)	24.30 ± 0.41^b^	24.49 ± 1.30^b^	33.04 ± 1.42^a^	<0.01
GR (U/g prot)	0.013 ± 0.001^b^	0.014 ± 0.001^b^	0.018 ± 0.001^a^	0.02
**Colon**				
T-AOC (U/mg prot)	0.16 ± 0.02^b^	0.27 ± 0.00^a^	0.25 ± 0.02^a^	0.01
MDA (nmol/mg prot)	2.75 ± 0.33	2.06 ± 0.09	2.28 ± 0.07	0.07
CAT (U/mg prot)	19.30 ± 3.70^b^	26.06 ± 0.41^ab^	28.51 ± 1.22^a^	0.03
T-SOD (U/mg prot)	735.12 ± 34.90	730.53 ± 25.70	763.99 ± 32.90	0.72
GSH-PX (U/mg prot)	11.27 ± 1.41^b^	12.11 ± 0.93^b^	16.23 ± 0.90^a^	0.02
GR (U/g prot)	0.010 ± 0.001^b^	0.014 ± 0.001^a^	0.017 ± 0.002^a^	<0.01

*Data were shown as means ± SE. n = 5 in each group. Control, basal diet; Na_2_SeO_3_, 0.3 mg Se/kg Na_2_SeO_3_; HMSeBA, 0.3 mg Se/kg HMSeBA. ^a,b,c^Mean values within a row with different superscript letters were significantly different (p < 0.05). T-AOC, total antioxidant capacity; MDA, malondialdehyde; CAT, catalase; T-SOD, total superoxide dismutase; GSH-Px, glutathione peroxidase; GR, glutathione reductase*.

### Effects of Organic Selenium on the Expression of Inflammatory Response Related Genes in Gilts

**Figure 3:**
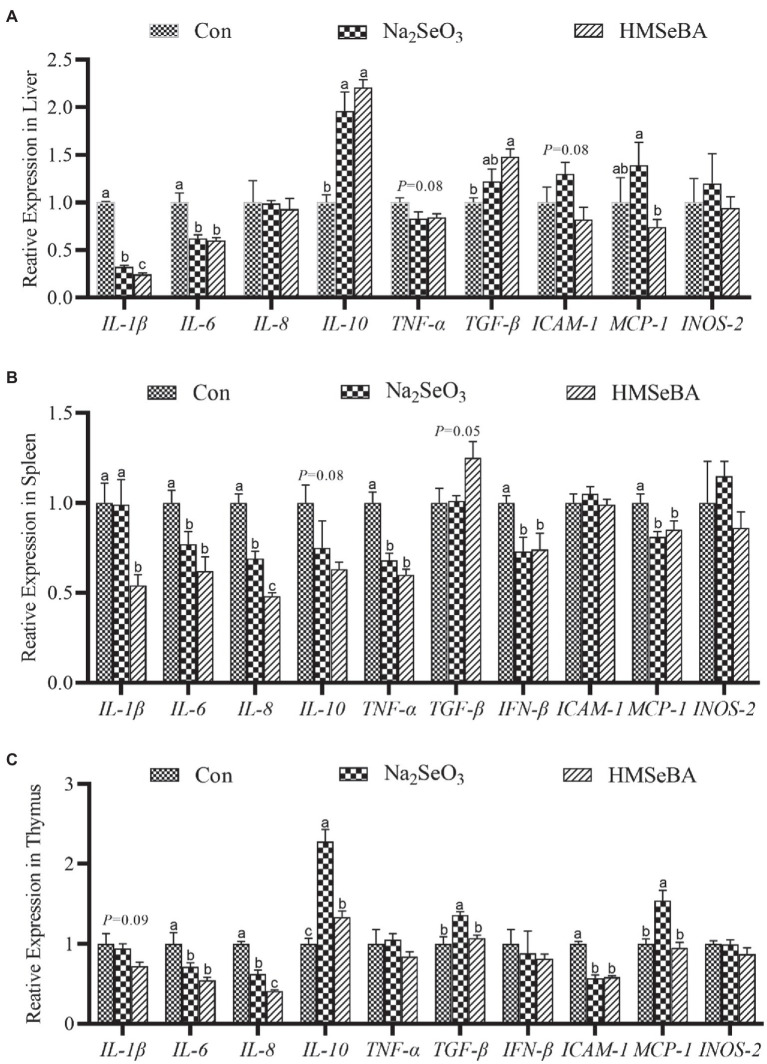
The effect of HMSeBA on the expression of related cytokines in the liver **(A)**, spleen **(B)**, and thymus **(C)** of gilts. *n* = 5 in each group. Data were shown as means ± SE. *n* = 5 in each group. Control, basal diet; Na_2_SeO_3_, 0.3 mg Se/kg Na_2_SeO_3_; HMSeBA, 0.3 mg Se/kg HMSeBA. ^a,b,c^Mean values within a row with different superscript letters were significantly different (*p* < 0.05). IL-1β, interleukin-1β; IL-6, interleukin-6; IL-8, interleukin-8; IL-10, interleukin-10; TNF-α, tumor necrosis factor-α; TGF-β, transforming growth factor-β; IFN-β, interferon-β; ICAM-1, intercellular cell adhesion molecule-1; MCP-1, monocyte chemotactic protein-1; INOS-2, inducible nitric oxide synthase-2.

The results showed that compared with the control and Na_2_SeO_3_ groups, the expression of *interleukin-1β* (*IL-1β*) and *IL-6* in the liver of gilts fed HMSeBA was significantly reduced (*p* < 0.05), while the expression of *interleukin-10* (*IL-10*) and *transforming growth factor-β* (*TGF-β*) increased (*p* < 0.05), and the expression of *TNF-α* and *intercellular cell adhesion molecule-1* (*ICAM-1*) had a decreasing trend (*p* = 0.08; Figure 3A). In the spleen, the expression of *interleukin-8* (*IL-8*) in the HMSeBA fed group was lower than that of the control and Na_2_SeO_3_ groups (*p* < 0.05). Compared with the control, the expression of *IL-1β*, *IL-6*, *TNF-α*, *IFN-β*, and *monocyte chemotactic protein-1* (*MCP-1*) in the HMSeBA group was significantly decreased (*p* < 0.05), and there was no difference from the Na_2_SeO_3_ group. The expression of *IL-10* and *TGF-β* showed a downregulated and upregulated trend, respectively, in the HMSeBA group (*p* = 0.08, *p* = 0.05; Figure 3B). Dietary supplementation with HMSeBA reduced the expression of *IL-8* (*p* < 0.05; Figure 3C) in the thymus compared with the control and Na_2_SeO_3_ groups. In addition, compared with the control group, the expression of *IL-10* in the HMSeBA group was increased (*p* < 0.05), the expression of *IL-6* and *ICAM-1* was decreased (*p* < 0.05), and the expression of *IL-1β* had a decreasing trend (*p* = 0.09).

**Figure 4:**
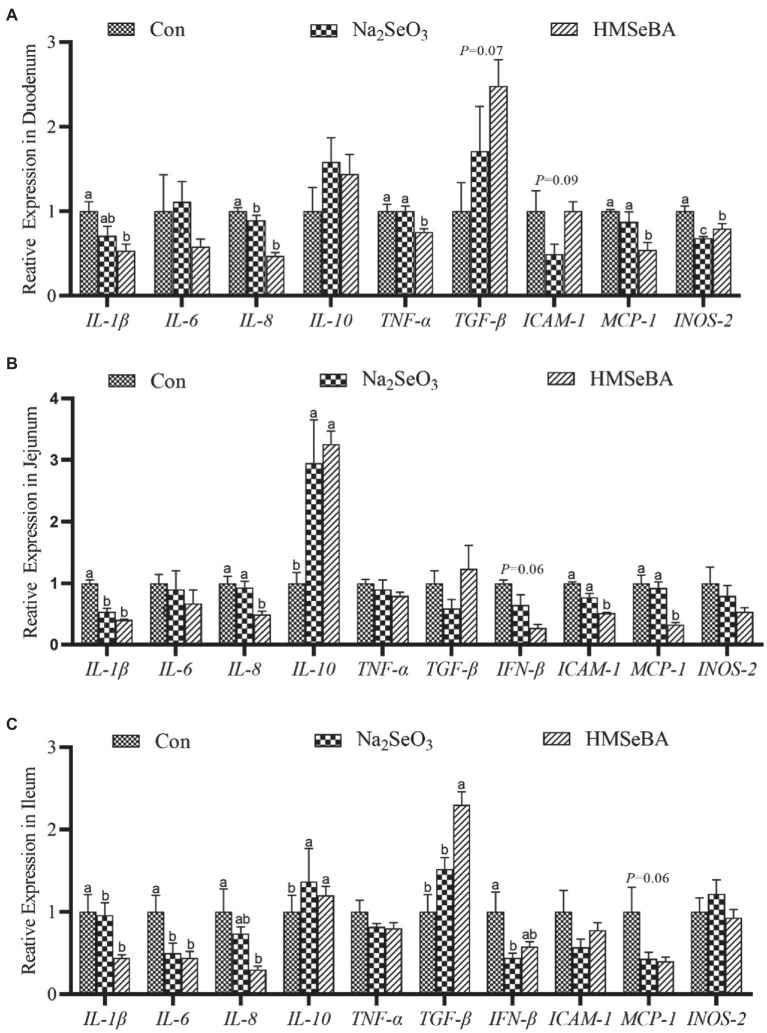
The effect of HMSeBA on the expression of related cytokines in duodenum **(A)**, jejunum **(B)**, and ileum **(C)** of gilts. *n* = 5 in each group. Data were shown as means ± SE. *n* = 5 in each group. Control, basal diet; Na_2_SeO_3_, 0.3 mg Se/kg Na_2_SeO_3_; HMSeBA, 0.3 mg Se/kg HMSeBA. ^a,b,c^Mean values within a row with different superscript letters were significantly different (*p* < 0.05). IL-1β, interleukin-1β; IL-6, interleukin-6; IL-8, interleukin-8; IL-10, interleukin-10; TNF-α, tumor necrosis factor-α; TGF-β, transforming growth factor-β; IFN-β, interferon-β; ICAM-1, intercellular cell adhesion molecule-1; MCP-1, monocyte chemotactic protein-1; INOS-2, inducible nitric oxide synthase-2.

The expression of *TNF-α* and *MCP-1* in the duodenum of gilts in the HMSeBA fed group was lower than those in the control and Na_2_SeO_3_ groups (*p* < 0.05; Figure 4A). In addition, the expression levels of *IL-1β*, *IL-8*, and *INOS-2* in the HMSeBA group were decreased (*p* < 0.05), and the expression of *TGF-β* tended to increase (*p* = 0.09) when compared with the control group (Figure 4A). Compared with the control and Na_2_SeO_3_ groups, the expression of *IL-8*, *ICAM-1*, and *MCP-1* in the jejunum of the HMSeBA fed group was decreased (*p* < 0.05), and the expression of *IL-10* was significantly increased (*p* < 0.05), while the expression of *IFN-β* had a decreasing trend (*p* = 0.06; Figure 4B). Compared with the control group, the expression of *IL-1β* in the HMSeBA and Na_2_SeO_3_ fed groups was significantly decreased (*p* < 0.05). In the ileum of gilts, the expression of *TGF-β* in the HMSeBA fed group was higher than that in the control and Na_2_SeO_3_ groups (*p* < 0.05), and the expression of *IL-1β*, *IL-6*, and *IL-8* was decreased (*p* < 0.05), the expression of *IL-10* was increased (*p* < 0.05), compared with the control group. The expression of *MCP-1* in the HMSeBA group had a decreasing trend (*p* = 0.06; Figure 4C).

### Effect of Organic Selenium on the Inflammatory Factors and Immunoglobulins in Gilts

Serum IL-2 and IgG concentrations in the HMSeBA group were higher than those in the control and Na_2_SeO_3_ groups (*p* < 0.05). In addition, adding HMSeBA significantly reduced serum IL-6 and TNF-α levels compared with the control group (*p* < 0.05), while TNF-α levels showed no significant difference compared with the Na_2_SeO_3_ group (Table 7). In the duodenum, jejunum, and ileum, HMSeBA improved the intestinal sIgA level compared with the control group (*p* < 0.05; Table 8), while there is no significant difference in the duodenal sIgA level compared with the Na_2_SeO_3_ group.

**Table 7:** Effect of HMSeBA on serum immunity indexes and immunoglobulins of gilt.

Item	Treatment			Values of *p*
	Control	Na_2_SeO_3_	HMSeBA	
IL-2, pg/ml	284.95 ± 11.91^b^	227.83 ± 11.75^c^	339.88 ± 12.24^a^	<0.01
IL-6, pg/ml	48.69 ± 2.53^a^	21.59 ± 1.84^b^	20.36 ± 0.56^b^	<0.01
IgA, μg/ml	98.29 ± 2.26	100.05 ± 3.55	105.52 ± 3.25	0.26
IgG, μg/ml	559.9 ± 4.24^b^	562.27 ± 6.96^b^	670.18 ± 6.39^a^	<0.01
IgM, μg/ml	137.89 ± 2.76	140.20 ± 1.36	145.29 ± 2.34	0.10
TNF-α, pg/ml	579.55 ± 20.63^a^	517.54 ± 30.07^ab^	462.26 ± 10.57^b^	<0.01

*Data were shown as means ± SE. n = 5 in each group. Control, basal diet; Na_2_SeO_3_, 0.3 mg Se/kg Na_2_SeO_3_; HMSeBA, 0.3 mg Se/kg HMSeBA. ^a,b,c^Mean values within a row with different superscript letters were significantly different (p < 0.05). IL-2, Interleukin-2; IL-6, Interleukin-6; IgA, immunoglobulin A; IgG, immunoglobulin G; IgM, immunoglobulin M; TNF-α, tumor necrosis factor-α*.

**Table 8:** The effect of HMSeBA on the concentration of sIgA in the intestines of gilts.

Item	Treatment			Values of *p*
	Control	Na_2_SeO_3_	HMSeBA	
Duodenum, μg/ml	4.45 ± 0.71^b^	6.92 ± 0.52^a^	8.21 ± 0.28^a^	<0.01
Jejunum, μg/ml	3.59 ± 0.68^b^	4.08 ± 0.59^b^	7.83 ± 0.43^a^	<0.01
Ileum, μg/ml	2.38 ± 0.41^b^	2.58 ± 0.29^b^	5.14 ± 0.43^a^	<0.01

*Data were shown as means ± SE. n = 5 in each group. Control, basal diet; Na_2_SeO_3_, 0.3 mg Se/kg Na_2_SeO_3_; HMSeBA, 0.3 mg Se/kg HMSeBA. ^a,b,c^Mean values within a row with different superscript letters were significantly different (p < 0.05). sIgA, secretory immunoglobulin A*.

### Organic Selenium Supplementation Changed the Intestinal Microbiota in Gilts

The results showed that there were 10 phylum relative abundances >0.1% in colon of gilts, included Firmicutes, Bacteroidetes, Spirochaetes, Actinobacteria, Proteobacteria, Euryarchaeota, Tenericutes, unidentified_Bacteria, and Melainabacteria (Table 9). Compared with the control group, the HMSeBA group increased the abundance of Firmicutes and the ratio of Firmicutes/Bacteroides (*p* < 0.05), while the abundance of Bacteroides, Melainabacteria, and Spirobacteria was reduced (*p* < 0.05).

**Table 9:** Effect of HMSeBA on the relative abundances at phyla level of colonic microbiota in gilts (%).

Item	Treatment			Values of *p*
	Control	Na_2_SeO_3_	HMSeBA	
Firmicutes	67.31 ± 3.49^b^	78.34 ± 1.29^a^	81.84 ± 1.45^a^	<0.01
Bacteroidetes	24.07 ± 2.82^a^	14.03 ± 0.72^b^	12.71 ± 1.37^b^	<0.01
Spirochaetes	4.42 ± 1.26	2.23 ± 0.16	2.09 ± 0.23	0.08
Actinobacteria	1.12 ± 0.28	2.43 ± 0.76	1.04 ± 0.07	0.11
Proteobacteria	1.24 ± 0.43	0.97 ± 0.25	0.55 ± 0.04	0.27
Euryarchaeota	0.34 ± 0.05	0.33 ± 0.12	0.17 ± 0.06	0.31
Tenericutes	0.36 ± 0.03	0.84 ± 0.23	1.04 ± 0.26	0.14
unidentified_Bacteria	0.10 ± 0.04	0.57 ± 0.23	0.17 ± 0.03	0.25
Melainabacteria	0.07 ± 0.00^a^	0.04 ± 0.00^b^	0.04 ± 0.00^b^	0.04
Others	0.14 ± 0.06	0.23 ± 0.05	0.28 ± 0.08	0.32
Firmicutes/Bacteroidetes	3.09 ± 0.64^b^	5.66 ± 0.39^a^	6.74 ± 0.70^a^	<0.01

*Data were shown as means ± SE. n = 5 in each group. Control, basal diet; Na_2_SeO_3_, 0.3 mg Se/kg Na_2_SeO_3_; HMSeBA, 0.3 mg Se/kg HMSeBA. ^a,b,c^Mean values within a row with different superscript letters were significantly different (p < 0.05)*.

The 35 most abundant genera in all samples were detected. Compared with the control group, the gilts supplemented with HMSeBA increased the abundance of *Terrisporobacter* and *Intestinibacter* (*p* < 0.05; Table 10), and decreased the abundance of *Prevotellaceae* and *Megasphaera* (*p* < 0.05). There was also an increasing trend in the abundance of *Phascolarctobacterium* (*p* = 0.07), while a decreasing trend in the abundance of *Parabacteroides* (*p* = 0.09).

**Table 10:** Effect of HMSeBA on the relative abundances at genera level of colonic microbiota in gilts (%).

Item	Treatment			Values of *p*
	Control	Na_2_SeO_3_	HMSeBA	
*Streptococcus*	22.61 ± 6.26	17.54 ± 3.80	22.17 ± 5.03	0.75
*Lactobacillus*	4.75 ± 2.41	4.80 ± 0.37	7.82 ± 2.24	0.50
*unidentified_Clostridiales*	8.46 ± 0.94	9.26 ± 1.98	8.81 ± 0.46	0.92
*Terrisporobacter*	3.69 ± 0.25^b^	5.92 ± 0.69^ab^	8.12 ± 1.26^a^	0.01
*unidentified_Ruminococcaceae*	2.32 ± 0.16^b^	4.75 ± 0.92^a^	3.11 ± 0.50^ab^	0.04
*Turicibacter*	2.17 ± 0.28	2.40 ± 0.31	3.49 ± 0.65	0.12
*Bifidobacterium*	0.65 ± 0.263	1.65 ± 0.68	0.45 ± 0.08	0.14
*Romboutsia*	1.22 ± 0.21	1.26 ± 0.20	1.54 ± 0.29	0.59
*Methanobrevibacter*	0.33 ± 0.05	0.33 ± 0.12	0.17 ± 0.06	0.32
*unidentified_Spirochaetaceae*	1.00 ± 0.35	0.78 ± 0.18	0.62 ± 0.08	0.52
*unidentified_Prevotellaceae*	1.38 ± 0.17^a^	1.06 ± 0.12^ab^	0.79 ± 0.10^b^	0.03
*unidentified_Lachnospiraceae*	0.93 ± 0.13	1.27 ± 0.16	1.10 ± 0.11	0.24
*Alloprevotella*	0.45 ± 0.09	0.48 ± 0.02	0.33 ± 0.02	0.23
*Mitsuokella*	0.27 ± 0.07	0.67 ± 0.21	0.35 ± 0.08	0.12
*Succinivibrio*	0.02 ± 0.00	0.03 ± 0.00	0.04 ± 0.01	0.12
*Megasphaera*	0.15 ± 0.03^b^	0.53 ± 0.05^a^	0.25 ± 0.04^b^	<0.01
*Phascolarctobacterium*	0.22 ± 0.02	0.34 ± 0.03	0.32 ± 0.04	0.07
*Anaerovibrio*	0.65 ± 0.15	0.67 ± 0.14	0.61 ± 0.09	0.96
*unidentified_Enterobacteriaceae*	0.15 ± 0.07	0.10 ± 0.03	0.11 ± 0.02	0.70
*Oscillospira*	0.25 ± 0.02	0.52 ± 0.14	0.45 ± 0.04	0.14
*Acetitomaculum*	0.20 ± 0.02^b^	0.52 ± 0.13^a^	0.26 ± 0.04^b^	0.04
*Blautia*	0.15 ± 0.02	0.16 ± 0.02	0.12 ± 0.01	0.39
*Oscillibacter*	0.24 ± 0.01	0.34 ± 0.06	0.33 ± 0.03	0.25
*Parabacteroides*	0.37 ± 0.07	0.21 ± 0.04	0.26 ± 0.02	0.09
*Lachnospira*	0.15 ± 0.02	0.15 ± 0.00	0.16 ± 0.01	0.89
*Intestinibacter*	0.16 ± 0.03^b^	0.25 ± 0.04^ab^	0.34 ± 0.05^a^	0.03
*Others*	44.76 ± 4.46	36.08 ± 1.90	33.91 ± 2.92	0.09

*Data were shown as means ± SE. n = 5 in each group. Control, basal diet; Na_2_SeO_3_, 0.3 mg Se/kg Na_2_SeO_3_; HMSeBA, 0.3 mg Se/kg HMSeBA. ^a,b,c^Mean values within a row with different superscript letters were significantly different (p <0.05)*.

### Relationship Between Serum Cytokines and Intestinal Microbiota

**Figure 5:**
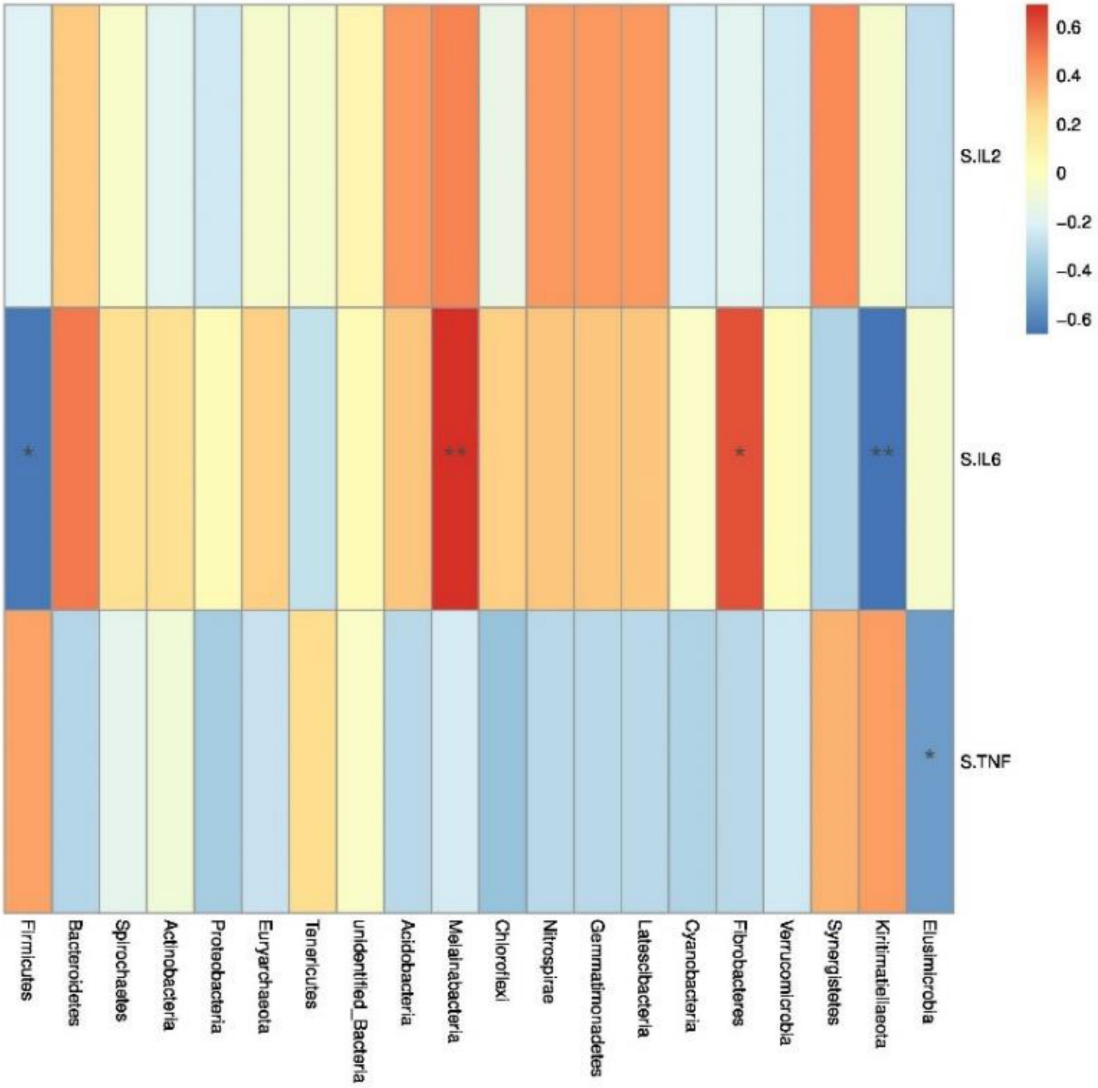
Heat map of correlation analysis between serum cytokines and microorganisms at the phylum level. S.IL2: serum IL-2; S.IL6: serum IL-6; S.TNF: serum TNF-α. *n* = 5. Spearman diagram showed that the ordinate was environmental factor information, and the abscess was species information. The value corresponding to the middle heat map was Spearman correlation coefficient r, which was between −1 and 1. *r* < 0 was negative correlation, *r*> was positive correlation, and the marker * indicated *p* < 0.05 for significance test.

Spearman correlation analysis was used to study the relationship between environmental factors and microbial species richness, to study the mutual change relationship between environmental factors and species. At the phylum level, Fibacteria and Melainabacteria were positively correlated with the serum cytokine IL-6 (*r* = 0.61, *p* < 0.05; *r* = 0.69, *p* < 0.05; Figure 5), Conversely, Kiritimatiellaeota and Firmicutes were negatively correlated with the serum cytokine IL-6 (*r* = −0.66, *p* < 0.05; *r* = −0.64, *p* < 0.05). Trachelum was negatively correlated with the serum cytokine TNF-α (*r* = −0.53, *p* < 0.05).

**Figure 6:**
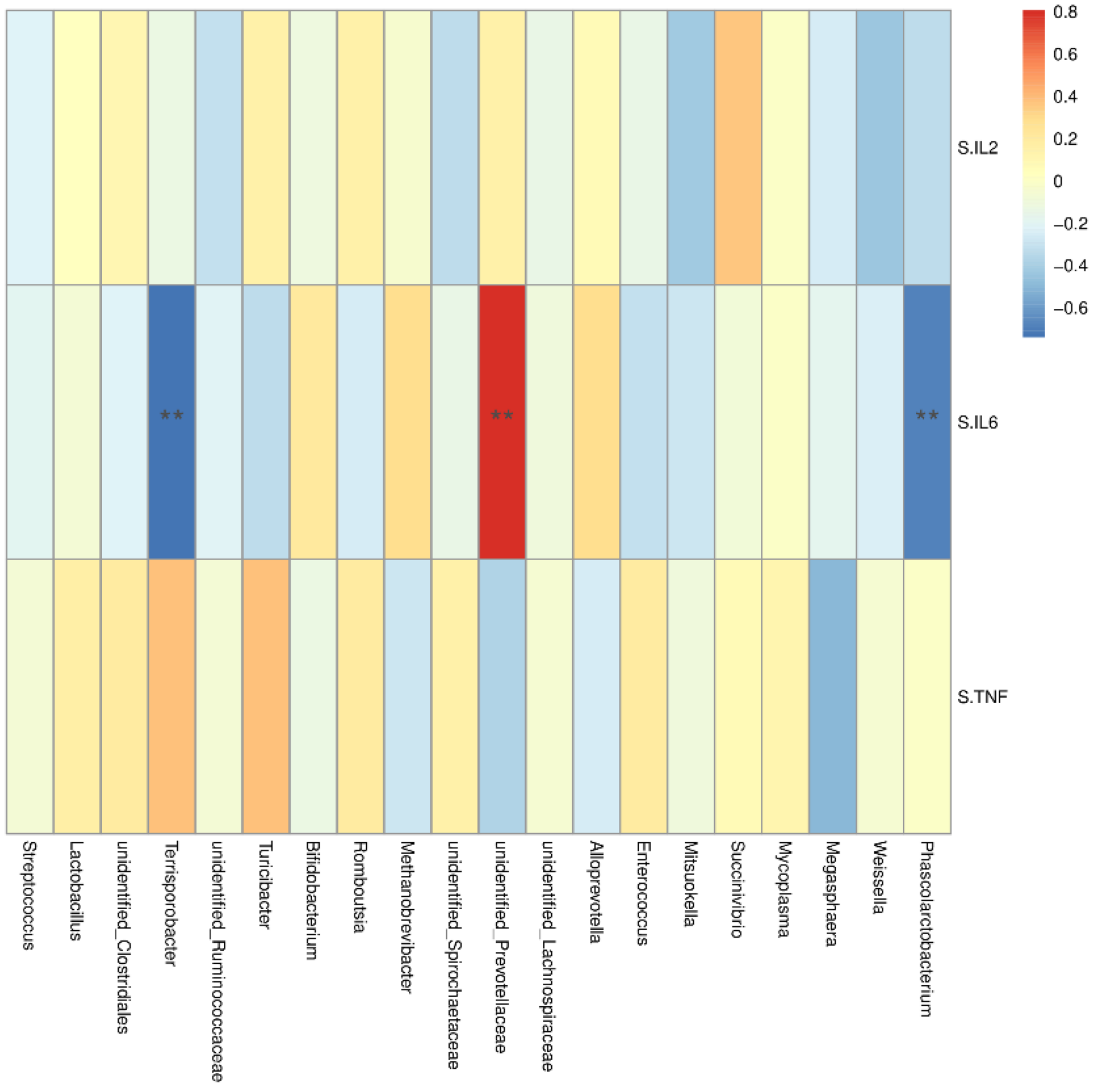
Heat map of correlation analysis between serum cytokines and microorganisms at the genus level. S.IL2: serum IL-2; S.IL6: serum IL-6 S.TNF: serum TNF-α. *n* = 5. Spearman diagram showed that the ordinate was environmental factor information, and the abscess was species information. The value corresponding to the middle heat map was Spearman correlation coefficient r, which was between −1 and 1. *r* < 0 was negative correlation, *r*> was positive correlation, and the marker * indicated *p* < 0.05 for significance test.

At the genus level, *Lacobacteria* and *Entomobacteria* were negatively correlated with the serum cytokine IL-6 (*r* = −0.68, *p* < 0.05; *r* = −0.74, *p* < 0.05; Figure 6), and *Prewolfella* was positively correlated with the serum cytokine IL-6 (*r* = 0.81, *p* < 0.05).

## Discussion

As an important part of intensive pig farms, reserve gilts are critical for both maintenance and expansion. Gilt reproduction performance is directly related to the overall level of pig production and the future of pig farms. To our knowledge, this is the first study to add HMSeBA to the basic diet of reserve gilts in order to study its effects on antioxidant capacity, immune function, and intestinal microflora. These results of current study can provide data reference and theoretical support for the application of HMSeBA in gilts, and it also have great significance to further improve the level of pig production in China, improve the breeding benefits of pig production, and improve the food composition of people.

In a general way, the level of selenium in animals mainly depends on the content of selenium in diet, and the absorption efficiency of organic selenium has proven to be superior to inorganic selenium (Juniper et al., 2008). Mou added HMSeBA to the basal diet of sows during pregnancy and lactation and found significantly increased plasma selenium concentrations in the sows and their offspring (Mou et al., 2020b). The present results showed that adding sodium selenite and HMSeBA to the diet significantly increased the selenium content in the tissues of the gilts, which was consistent with previous studies in pigs (Speight et al., 2012), poultry (Briens et al., 2013), and sheep (Faixová et al., 2016). The present study also found that the organic selenium HMSeBA significantly increased the selenium content in several tissues compared to inorganic selenium. The difference in the deposition efficiency of organic selenium and inorganic selenium may be related to their different absorption and metabolic pathways. Inorganic selenium is absorbed by animals in a passive manner and reduced to selenium by TrxR or by reaction with glutathione (Daniels, 1996). While organic selenium is actively absorbed through amino acid transport mechanisms whereby organic selenium compounds are converted to selenocysteine through the antiselenylation pathway, and selenocysteine generates selenides under the action of selenocysteine lyase (Fairweather-Tait et al., 2010; Mehdi et al., 2013; Burk and Hill, 2015; Shini et al., 2015). SelP1 is one of the main selenoproteins in plasma, which is composed of up to 10 selenocysteine residues and is very sensitive to changes of selenium levels. And the main function of SelP1 is to transport selenium to various tissues, selenocysteine produces selenide under the action of se cysteine lyase (Burk and Hill, 2009). In this study, the HMSeBA molecule contains 40% Se and is higher than inorganic selenium. It can be converted to selenocysteine more quickly and is rarely lost to the excretion pathway. Therefore, HMSeBA can be better absorbed and utilized by the gilts, leading to the selenium deposition effect in the tissues being more significant, and compared with control group the expression of *SelP1* in the tissues of the HMSeBA group gilts increasing.

The main antioxidant enzyme in the body is GSH-Px, whose activity will affect the level of reactive oxygen free radicals and the content of malondialdehyde and the final product of lipid peroxidation in the body. Selenium is a component of this enzyme and located in its active center, so the selenium status of the body directly affects the activity of GSH-Px (Brigelius-Flohé et al., 2003; Wang et al., 2011; Mistry et al., 2012). Mou et al. (2020b) fed organic selenium to pregnant sows and found this significantly increased the activity of GSH-Px in the serum and placenta of the sows, and significantly decreased the content of MDA (Mou et al., 2020b). Other studies showed that the activity of GSH-Px in the duodenum, jejunum, and rectum of chicks fed Se-deficient diets were significantly lower than those in basal diets (Ruan et al., 2018). The present study showed that HMSEBA significantly increased the activities of GSH-Px, T-SOD, and CAT in the thymus, jejunum, ileum, and colon of gilts, and the content of MDA in each tissue showed a decreasing trend. In this study, HMSEBA not only improved the antioxidant capacity of gilts, but also significantly upregulated the gene expressions of *GPX1-4* in the spleen and thymus of the gilts, as well as *GPX1*, *GPX3*, and *GPX4* in the intestinal tract of the gilts. Selenium functions mainly through selenoprotein, and *GPX1-4* plays an important role in regulating oxidative stress and inflammation in the intestinal tract (Yan and Chen, 2006; Lubos et al., 2010; Avery and Hoffmann, 2018). *GPX2* has been reported to inhibit cox-dependent PGE2 production, suggesting that *GPX2* has a potential anti-inflammatory effect within the gastrointestinal tract (Kipp et al., 2007; Banning et al., 2008). Deletion of the *GPX1* and *GPX2* genes in mice led to severe inflammation in the form of spontaneous colitis (Esworthy et al., 2011). In addition, the downregulation of *GPX3* and *GPX4* expression can also induce severe colitis and enhance tumorigenesis (Speckmann et al., 2011; Barrett et al., 2013). Therefore, supplementing selenium can upregulate the expression of *GPX*, thus preventing oxidative damage in the gastrointestinal tract and preventing inflammation.

Rats and cell culture experiments have reported that downregulation of *TrxR1* and *TrxR2* in response to selenium deficiency affects immune organs and then alters cellular signaling pathways regulated by redox, leading to increased inflammation (Flohé et al., 1997; Hirota et al., 1997; Lothrop et al., 2014). Moreover, the Ca^2+^ dependent function of SelK-deficient mice cells was decreased, and the pro-inflammatory cytokines *IL-6*, *MCP-1*, and *TNF-α* were significantly increased, while the expression of pro-inflammatory cytokines was downregulated in mice with normal *SelK* content (Nelson et al., 2011). It has been found that selenium supplementation downregulates the expression of inflammatory factors in cultured cells, and that a good selenium status can maintain the immune system under both infection and inflammatory conditions (Kudva et al., 2015). The present results showed that dietary supplementation with HMSeBA in the gilt, raised the thymus *TrxR1*, jejunum, ileum *TrxR1* and *TrxR2*, and all tissues (except the thymus) *SelK* expression, and reduced the spleen, thymus, and intestinal tissue of proinflammatory factor (*IL-1β*, *IL-6*, *TNF-α*, *IFN-β*, *MCP-1*, *ICAM-1*, and *INOS-2*) expression, increased the level of anti-inflammatory cytokines *IL-10* and the expression of *TGF-β*. Tsuji et al. also obtained similar results, by increasing selenium level in the diet, the expression levels and translation of stress-related selenoproteins *TrxR1* and *TrxR2* mRNA, as well as the expression levels and translation of genes related to inflammation and interferon-γ reaction increased, thus reduced the inflammatory response of the body and improved the immune function (Verma et al., 2011). We also found that HMSEBA significantly upregulated the expression of SEPHS2 in each intestinal segment of gilts, and SEPHS2 was mainly used as a catalyst to produce selenium phosphate and participate in the synthesis of all selenoproteins (Xu et al., 2007). Therefore, other selenoproteins in the intestine were upregulated because of the upregulated expression of *SEPHS2*, thus reducing the level of inflammation in the body and improving the immune function.

It can be seen from the above results that the immune system relies on a good selenium state to fight bacterial and viral infections, deal with oxidative damage, and regulate inflammation. It has been reported that adding selenium in the basal diet of sows effectively increased the serum IgA, IgG, and IgM concentrations of sows and their offspring (Gelderman and Clapper, 2014). When adding inorganic selenium or organic selenium to the diet, the serum IgG concentration of the ewe increased (Cabello et al., 1983). Low selenium content may also affect intestinal mucosal immunity. In commercial broilers, selenium deficiency reduced the content of soluble IgA in the duodenal mucosa and increased the level of pro-inflammatory cytokine IL-1β. In contrast, anti-inflammatory cytokines, such as TGF-β1 and IL-10, were significantly inhibited (Liu et al., 2016). In present study, the HMSeBA group increased the protein concentrations of IL-2 and IgG in serum. And compared with the control group, the HMSeBA group decreased the protein concentrations of proinflammatory factors IL-6 and TNF-α, and increased the concentration of sIgA in the intestine. Studies have shown that low selenium leads to reduce intestinal sIgA secretion, a lower intestinal immunological barrier, and thus reduces intestinal immune function (Liu et al., 2016). The reason may be that selenium deficiency causes the increase of free radicals in the intestinal mucosa, which increases the expression of inflammatory factors, and leads to intestinal microcirculation disorders, an imbalance of T cells, blocked proliferation of lymphocytes associated with the intestine, and leads to the decrease of intestinal sIgA secretion (Hanson, 1998).

The intestinal microbiota can be regulated by dietary supplements that have the ability to stimulate the growth of beneficial bacteria and selectively inhibit the activity of pathogenic bacteria (Hrdina et al., 2009). Molan et al. found that compared with the extract without selenium, selenium extract significantly increased the number of Lactobacillus and Bifidobacterium in the cecum of rats, and reduced the number of *Escherichia coli* and Salmonella (Molan et al., 2010). The present results showed that dietary supplementation with HMSeBA reduced the number of Parabacteroides and Prevotellaceae in the intestine, and increased the number of Ruminococcaceae and Phascolarctobacterium. At the phylum level correlation analysis, Firmicutes was negatively correlated with serum levels of cytokines, 16S rRNA results showed that the HMSeBA treatment group increased the abundance of Firmicutes in the colon chyme, indicating that HMSeBA could downregulate the level of inflammatory cytokines. Meanwhile, inflammatory factor levels may also affect the abundance of microbiota, thus affecting the overall diversity of microbial communities. Dietary selenium can affect the overall diversity of existing intestinal microbiota as well as the establishment of gastrointestinal microbiota, and the Parabacteroides group of Bacteroides is inversely associated with selenium supplementation. This finding might be explained by the use of selenium by various microorganisms and the toxicity of selenium to some organisms (Kasaikina and Kravtsova, 2011). The sensitivity of microorganisms to dietary selenium may be related to the regulation of host selenium status, and it has been shown that gastrointestinal microbiota affects host selenium status and selenium protein expression. Under the condition of selenium deficiency, the activities of GPX and TR in liver and intestine of germ-free (GF) mice were higher than those of conventionalized (CV) mice, and the expression of GPX1 and its mRNA in liver and colon were also higher (Hrdina et al., 2009). These results were consistent with marina’s study, which found that the level of selenoprotein in GF mice was higher than that in CV mice (Fukushima et al., 2003). Therefore, dietary selenium affects the host’s selenium status and the expression of selenoproteins by affecting the composition of intestinal flora, and then affects the level of inflammatory factors and immune function.

## Conclusion

In conclusion, adding 0.3 mg Se/kg HMSeBA to the diet can improve tissue selenium content, antioxidant capacity, immunoglobulin concentration, and immune-related selenoprotein gene expression of gilts, reduce the level of proinflammatory factors, promote the growth of intestinal beneficial bacteria, and further enhance the immune function of gilts.

## Data Availability Statement

The datasets generated for this study can be found in online repositories. The names of the repository/repositories and accession number(s) can be found at: https://www.ncbi.nlm.nih.gov/, PRJNA750710.

## Ethics Statement

The animal study was reviewed and approved by All animal procedures used in this study were approved by the Animal Care and Use Committee of Sichuan Agricultural University. Written informed consent was obtained from the owners for the participation of their animals in this study.

## Author Contributions

SX, ZL, and DW designed the study. ZL and YD carried out the animal experiments and performed the laboratory work. ZL, YD, SC, XJia, XJian, LC, YL, JL, BF, ZF, YZ, JW, and HX performed the statistical analysis. ZL wrote the paper. SX and DW revised the manuscript. All authors contributed to the article and approved the submitted version.

## Conflict of Interest

The authors declare that the research was conducted in the absence of any commercial or financial relationships that could be construed as a potential conflict of interest.

## Publisher’s Note

All claims expressed in this article are solely those of the authors and do not necessarily represent those of their affiliated organizations, or those of the publisher, the editors and the reviewers. Any product that may be evaluated in this article, or claim that may be made by its manufacturer, is not guaranteed or endorsed by the publisher.

## Footnotes

^1^ http://drive5.com/uparse/

^2^ http://github.com/rdpstaff/

^3^ http://www.arb-silva.de/
